# Prevalence of Fatigue, Risk Factors, and Relationship With Self-Rated Health Six Months After ICU Discharge in Japan: An Ambidirectional Cohort Study

**DOI:** 10.7759/cureus.76879

**Published:** 2025-01-03

**Authors:** Sachi Niiyama, Takeshi Unoki, Junpei Haruna, Hiroomi Tatsumi, Yoshiki Masuda

**Affiliations:** 1 Department of Advanced Critical Care and Emergency Center, Sapporo Medical University Hospital, Sapporo, JPN; 2 Department of Acute and Critical Care Nursing, School of Nursing, Sapporo City University, Sapporo, JPN; 3 Department of Intensive Care Medicine, Sapporo Medical University School of Medicine, Sapporo, JPN

**Keywords:** critical care, fatigue, intensive care unit, post-intensive care syndrome, survivors

## Abstract

Background

Fatigue presents an important challenge for patients discharged from intensive care units (ICUs). Despite its importance, data on the prevalence and clinical impact of post-ICU fatigue remain limited. In particular, the proportion of patients in clinical settings in Japan who find fatigue distressing, as well as the associations between fatigue, risk factors, and self-rated health, have not been fully explored using validated fatigue measures. Therefore, this study aimed to assess the prevalence of fatigue, identify its associated risk factors, and examine the relationship between fatigue and self-rated health status six months after ICU discharge in Japan.

Methods

This single-center, ambidirectional cohort study administered a survey to patients aged ≥18 years, six months after ICU discharge, to assess fatigue and self-rated health. Retrospective data were also collected from patients' medical records during hospitalization. Fatigue prevalence was the primary endpoint, measured using the cutoff value of the Functional Assessment of Chronic Illness Therapy-Fatigue (FACIT-F) scale. Logistic regression analysis was performed to identify risk factors for fatigue. Locally weighted scatterplot smoothing (LOWESS) plots were generated with FACIT-F scale scores, and the association between fatigue and EuroQol 5 Dimensions 5-Level Visual Analog Scale (EQ-VAS) was analyzed.

Results

Questionnaires were sent to 87 patients, of whom 81 (93.1%) responded. Eight patients (9.9%) with missing FACIT-F scores were excluded, leaving 73 patients for analysis. The median (interquartile range) age of eligible patients was 74 (63-81) years, and 33 patients (45%) were male. Forty-six patients (63%) reported fatigue. Multivariate analysis identified a higher body mass index (BMI) at ICU admission as an independent risk factor for fatigue (odds ratio (OR) = 1.195; 95% confidence interval (CI) 1.018-1.447; p < 0.05). EQ-VAS scores were significantly lower in the fatigue group (OR 72.5, 95% CI 60-83.8 vs. OR 90, 95% CI 80-90; p < 0.01).

Conclusion

This study found that many patients in Japanese ICUs experience fatigue, even when disease severity is low. Additionally, BMI at ICU admission was identified as an independent risk factor for fatigue six months after ICU discharge. Regular follow-up on fatigue after ICU discharge is essential for improving long-term outcomes.

## Introduction

Patients discharged from intensive care units (ICUs) are at risk for developing post-intensive care syndrome (PICS), which is characterized by physical, mental, and cognitive impairments. Since its recognition, PICS has been the subject of significant research efforts, highlighting its long-term impact on patients' quality of life (QOL). However, while substantial progress has been made in understanding the traditional domains of PICS, unresolved challenges remain, such as the under-recognition of symptoms such as fatigue [1]. Fatigue is a distressing and prevalent symptom among post-ICU patients [2]. Despite its importance, data on the prevalence and clinical impact of post-ICU fatigue remain limited [3]. Therefore, addressing this knowledge gap is crucial for improving post-discharge care and developing effective follow-up interventions. However, research on post-ICU fatigue has several limitations. First, the prevalence of fatigue among ICU survivors in Japan remains unclear. The prevalence and risk factors may differ in Japanese ICUs, which have a higher proportion of older patients [4], and cultural differences between Asian and Western populations may also play a role. Previous studies suggest that East Asian populations are more prone to psychological distress in interpersonal relationships than Western populations and may be more likely to experience fatigue [5]. Second, many studies investigating risk factors for fatigue have focused on specific patient groups, such as those with acute respiratory distress syndrome (ARDS) [6]. As a result, the identified risk factors may not fully represent those observed in the general ICU patient population. Third, few studies have explored the relationship between fatigue and self-rated health. Self-rated evaluations are crucial indicators of patients' perceived symptoms, providing valuable insights that may be challenging to capture through objective measures. Examining the relationship between fatigue and self-rated health is crucial for understanding whether fatigue negatively impacts perceived health status.

Given these considerations, the proportion of patients in clinical settings in Japan who find fatigue distressing, as well as the associations between fatigue, risk factors, and self-rated health, have not been fully explored using validated fatigue measures. Therefore, this study aimed to assess the prevalence of fatigue, identify its associated risk factors, and examine the relationship between fatigue and self-rated health six months after ICU discharge among medical-surgical patients in Japan. This approach has the potential to enhance our understanding of the challenges ICU survivors face due to fatigue and inform strategies for its prevention and management in the future.　

## Materials and methods

Study design

This ambidirectional cohort study was conducted in the 10-bed medical-surgical ICU at Sapporo Medical University Hospital. Data were collected using an opt-out survey, which included responses from both participants and nonrespondents. A survey questionnaire was sent to patients discharged from the ICU six months prior, to assess their current fatigue and self-rated health. Retrospective data were also collected from patients' medical records during hospitalization. We ensured that the patients understood the study description and consented to participate by signing and returning the consent form to the participating institutions. This study was approved by the Ethics Review Board of the Graduate School of Nursing of Sapporo City University (10 2023-10-13, Central IRB) and the Hospital Director of Sapporo Medical University Hospital (352-125 2023-10-23).

Participants and recruitment process

We retrospectively enrolled consecutive patients discharged from the ICU six months prior, based on medical record data. The inclusion criteria were patients aged ≥18 years who had been admitted to the ICU. The exclusion criteria included patients (1) with severe cognitive impairment or disorders of consciousness; (2) readmitted to the ICU or hospital within six months of discharge; (3) hospitalized or institutionalized at the time of the survey; (4) unable to be contacted by telephone; (5) who declined participation; (6) unable to complete a self-administered questionnaire; and (7) deemed unable to participate by their families. We included patients discharged between April 1 and September 30, 2023. We initially contacted patients by telephone to confirm eligibility and verify exclusion criteria. At this stage, formal informed consent was not obtained, but a brief explanation of the study was provided. If patients or their families did not respond to at least three telephone attempts on different days, they were recorded as "unable to contact." Once eligibility was confirmed, a survey package containing an explanatory leaflet, relevant questionnaires, and consent forms was mailed to each patient. Informed consent was obtained upon return of the signed consent form. If no response was received within two weeks, a reminder call was made. Survey data were collected from October 23, 2023, to March 31, 2024.

Data collection

The survey package included the Functional Assessment of Chronic Illness Therapy Fatigue (FACIT-F) scale [7] and the EuroQol 5-Dimensions 5-Level (EQ-5D-5L) questionnaire [8]. Additionally, the survey collected data on cohabitation, caregiver availability, employment status, and use of social support resources.

The primary outcome (fatigue prevalence) was assessed using the FACIT-F scale, a 13-item questionnaire designed to assess fatigue, originally developed for cancer patients to assess anemia-related symptoms. Previous studies have explored fatigue following ICU discharge using various scales, and this study used the FACIT-F scale, the reliability and validity of which have been validated, specifically in post-ICU patient populations. Among ICU survivors, the FACIT-F demonstrated strong reliability and validity, with a Cronbach's α of 0.937 and a correlation coefficient of 0.660 (p < 0.001) with the 36-Item Short Form Survey (SF-36) vitality scale [9]. We utilized the Japanese version of the FACIT-F scale, which was developed through item generation (translation and back-translation), pilot testing, and psychometric validation by Yoshimura et al. [10]. The Japanese version demonstrated a Cronbach’s α of 0.93 and significant correlations with Eastern Cooperative Oncology Group performance status ratings and FACIT-F scores (Spearman's rho = -0.48) [10]. We used this scale after registering and obtaining approval through the FACIT Group (https://www.facit.org/license-registration-form). The 13-item FACIT-F evaluates fatigue symptoms experienced over the previous seven days, with scoring on a five-point Likert scale ranging from "not at all" to "very much." Scores range from 0 (severe fatigue) to 52 (negligible fatigue), with lower scores indicating more severe fatigue. Raw scores were converted to a transformed scale (range, 0-100), with a score ≤ 68 indicating clinically significant fatigue compared to the general population [11].

The EQ-5D-5L is a validated and standardized tool used to measure health-related QOL [8]. We used the Japanese version of the EQ-5D-5L, which is available through the EuroQol Group (https://euroqol.org/). The EQ-5D-5L consists of five dimensions: mobility, self-care, usual activities, pain/discomfort, and anxiety/depression. Each dimension has five levels: no, slight, moderate, severe, and extreme problems. Health status is represented by 3,125 possible combinations, each of which is converted into a QOL score ranging from 0 (death) to 1 (perfect health), based on a Japanese value set [12]. The EQ-5D-5L also includes a Visual Analog Scale (VAS) ranging from 0 (worst imaginable health) to 100 (best imaginable health). This study used the EQ-5D-5L VAS (EQ-VAS) to evaluate self-rated health [13].

The demographic and clinical data of each patient were retrospectively collected from their medical records. These included variables such as age, sex, height, weight at ICU admission, body mass index (BMI), primary diagnosis, primary department, reason for ICU admission, Charlson Comorbidity Index [14], Acute Physiology and Chronic Health Evaluation II (APACHE II) score [15], medical history, use of circulatory assist devices, continuous renal replacement therapy, tracheostomy, history of surgery, duration of mechanical ventilation, length of hospital stay, length of ICU stay, and medications (sedative and analgesic exposure was defined as at least 24 hours of continuous intravenous infusion), as well as vasopressor and steroid use and patient outcomes.

Statistical analysis

Based on previous studies, the prevalence of fatigue after ICU discharge was estimated to be 70% [6], with a 95% confidence interval and an acceptable error of 0.10 [16]. To achieve an 80% response rate and an acceptable margin of error of 0.10, as informed by previous studies and considering clinically practical levels of precision, a sample size of 80 was required. Assuming an 80% response rate, we planned to administer the questionnaire to 100 patients.

Descriptive statistics were calculated for data analysis. The Shapiro-Wilk test was used to assess the normality of the data distribution. Means and standard deviations for normally distributed data were reported, while medians and interquartile ranges (IQRs) were provided for non-normally distributed data. Categorical variables were expressed as frequencies and percentages.

Fatigue, as measured by the FACIT-F scale cutoff, was compared across groups using the Mann-Whitney U test for continuous variables and Pearson's chi-square test for categorical variables. Logistic regression analysis was performed to identify risk factors for fatigue. Based on previous literature and clinical experience [2], covariates were selected, including age, sex, and variables that were significantly associated with the dependent variable (transformed FACIT-F ≤ 68) in the univariate analysis (p < 0.2) [17].

Locally weighted scatterplot smoothing (LOWESS) plots were generated with the FACIT-F scale scores, and the association between fatigue and EQ-VAS scores was analyzed.

In cases of missing data, if the missing data were not completely at random, missing values for each score were imputed using "multiple imputation by chained equations."

All analyses were performed using EZR (Saitama Medical Center, Jichi Medical University, Saitama, Japan) and R version 4.2.0 (R Foundation for Statistical Computing, Vienna, Austria). Two-sided significance tests were used for all analyses, with statistical significance set at p < 0.05.

## Results

**Figure 1:**
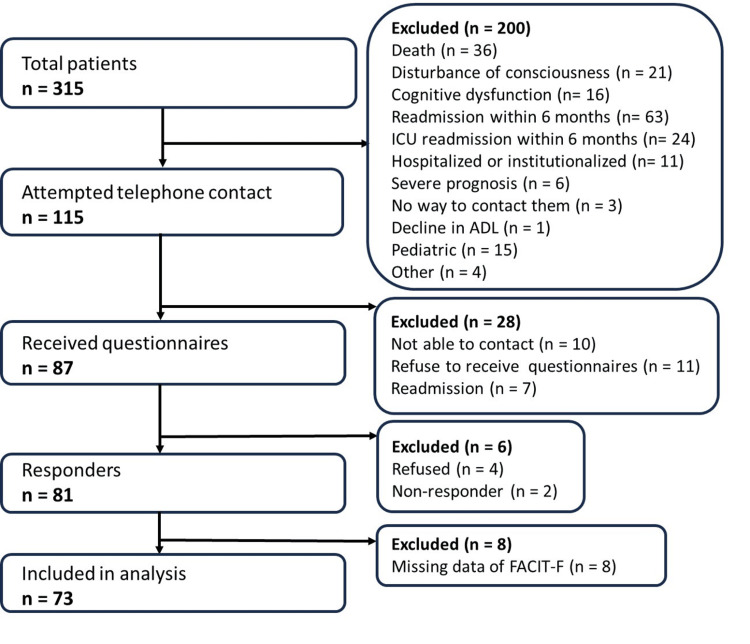
Flow diagram of patient enrollment FACIT-F: Functional Assessment of Chronic Illness Therapy-Fatigue, ADL: activities of daily living, ICU: intensive care unit.

A flow diagram of the study enrollment process is shown in Figure 1. Survey questionnaires were distributed to 87 enrolled patients, of whom six did not respond, resulting in 81 completed surveys (response rate, 93.1%). Among the respondents, eight (9.9%) patients had missing FACIT-F data and were therefore excluded from the analysis. This left 73 eligible patients. In this study, most of the missing data were related to the FACIT-F scale. Therefore, no imputation was performed for the missing data, and an available case analysis was employed.

The clinical characteristics of the patients are summarized in Table 1. The median age was 74 years (range, 63-81 years), and 33 patients (45%) were male. The median Acute Physiology and Chronic Health Evaluation II (APACHE II) score was 12 (8-20). The median ICU and hospital lengths of stay were two (2-3) and 22 (17-35) days, respectively.

**Table 1:** Patient characteristics Missing data: household composition = 1; caregiver = 1; social resource = 1; employment = 1. IQR: interquartile range, ICU: intensive care unit.

Variables	Values (N = 73)
Age (years), median (IQR)	74 (63-81)
Male, n (%)	33 (45)
Body mass index, kg/m^2^, median (IQR)	23.5 (21.3-25.1)
Primary department, n (%)	
Cardiovascular surgery	18 (25)
Cardiology	14 (19)
Gastrointestinal surgery	13 (18)
Orthopedic surgery	5 (7)
Otolaryngology	5 (7)
Neurosurgery	4 (5)
Respiratory surgery	3 (4)
Oral surgery	3 (4)
Obstetrics	3 (4)
Gynecology	2 (3)
Dermatology	1 (1)
Ophthalmology	1 (1)
Urology	1 (1)
Presence of cancer, n (%)	31 (42)
Presence of mental illness, n (%)	2 (3)
Reason for ICU admission, n (%)	
Elective surgery	55 (75)
Urgent	18 (25)
Acute Physiology and Chronic Health Evaluation Ⅱ score, median (IQR)	12 (8-20)
Charlson Comorbidity Index, median (IQR)	1 (0-2)
Duration of mechanical ventilation, median (IQR)	0 (0-2)
Length of ICU stay, median (IQR)	2 (2-3)
Length of hospital stay, median (IQR)	22 (17-35)
Treatment in ICU, n (%)	
Mechanical ventilation	33 (45)
Opioid	9 (12)
Vasopressor	7 (10)
Corticosteroid	11 (15)
Sedation	7 (10)
Intra-aortic balloon pumping	2 (3)
Continuous renal replacement therapy	2 (3)
Tracheostomy	2 (3)
Operation	67 (92)
Household composition, n (%)	
Living alone	14 (19)
With someone else	58 (81)
Caregiver, n (%)	61 (85)
Social resource, n (%)	7 (10)
Employment, n (%)	16 (22)
Outcome, n (%)	
Discharged home	62 (85)
Hospital transfer	11 (15)

The median FACIT-F score was 41 (range, 37-46), and the median transformed FACIT-F score was 66 (range, 62-72). Clinically significant fatigue (transformed FACIT-F ≤ 68) was reported by 46 patients (63%). Table 2 compares the clinical characteristics of patients with and without clinically significant fatigue (transformed FACIT-F ≤ 68). In the univariate analysis, BMI was higher in the group with clinically significant fatigue.

**Table 2:** Association between clinical characteristics and the presence of fatigue Missing data: household composition = 1; caregiver = 1; social resource = 1; employment = 1. IQR: interquartile range, ICU: intensive care unit.

Variables	Fatigue (n = 46)	Non-fatigue (n = 27)	p-value
Age (years), median (IQR)	74 (66.3-79.5)	73 (63-81.5)	0.882
Male, n (%)	19 (41)	14 (52)	0.538
Body mass index, kg/m^2^, median (IQR)	24 (22.2-25.4)	22 (20.3-24.2)	0.026
Primary department, n (%)			
Cardiovascular surgery	11 (24)	7 (26)	0.879
Respiratory surgery	2 (4)	1 (4)	
Gastrointestinal surgery	8 (17)	5 (19)	
Neurosurgery	3 (7)	1 (4)	
Orthopedic surgery	2 (4)	3 (11)	
Dermatology	0 (0)	1 (4)	
Otolaryngology	4 (9)	1 (4)	
Oral surgery	1 (2)	2 (7)	
Ophthalmology	1 (2)	0 (0)	
Urology	1 (2)	0 (0)	
Obstetrics	2 (4)	1 (4)	
Gynecology	1 (2)	1 (4)	
Cardiology	10 (22)	4 (15)	
Reason for ICU admission, n (%)			
Elective surgery	33 (72)	22 (81)	0.515
Urgent	13 (28)	5 (19)	0.515
Acute Physiology and Chronic Health Evaluation Ⅱ score, median (IQR)	12.5 (8-18)	11 (8.5-20)	0.864
Charlson Comorbidity Index, median (IQR)	1 (0-2)	1 (0-2)	0.233
Duration of mechanical ventilation, median (IQR)	0.5 (0-1.7)	0 (0-2)	0.541
Length of ICU stay, median (IQR)	2 (2-3)	2 (2-2.5)	0.625
Length of hospital stay, median (IQR)	20 (15-28.8)	25 (19.5-39)	0.087
Treatment in ICU (%)			
Mechanical ventilation	23 (50)	10 (37)	0.406
Opioid	5 (11)	4 (15)	0.9
Vasopressor	3 (7)	4 (15)	0.453
Corticosteroid	8 (17)	3 (11)	0.352
Sedation	4 (9)	3 (11)	1
Intra-aortic balloon pumping	1 (1)	1 (4)	1
Continuous renal replacement therapy	2 (4)	0 (0)	0.722
Tracheostomy	1 (1)	1 (4)	1
Operation	43 (46)	24 (89)	0.804
Presence of cancer	17(37)	14 (52)	0.318
Presence of mental illness	2 (4)	0 (0)	0.722
Household composition, n (%)			
Living alone	7 (15)	7 (26)	0.442
Living with someone else	38 (83)	20 (74)	0.442
Caregiver, n (%)	40 (87)	21 (78)	0.352
Social resource, n (%)	3 (7)	4 (15)	0.472
Employment, n (%)	8 (17)	8 (30)	0.38
Outcome, n (%)			
Discharged from home	38 (83)	24 (89)	0.7
Hospital transfer	8 (17)	3 (11)	0.7

Table 3 shows the results of the multivariate analysis used to identify the risk factors for fatigue. A high BMI at ICU admission was the only independent factor associated with fatigue.

**Table 3:** Risk factors for fatigue in multivariate analysis Cl: confidence interval.

Variables	Odds ratio	95%Cl	p-value
Male	1.39	0.501-3.886	0.525
Age	1.013	0.975-1.051	0.508
Length of hospital stay	0.986	0.954-1.018	0.375
Body mass index	1.195	1.018-1.447	0.047

Table 4 presents the EQ-VAS scores. The overall EQ-VAS was 80 ((IQR, 70-90). The EQ-VAS scores were significantly lower in the fatigue group compared to those without fatigue symptoms (72.5 (60-83.8) vs 90 (80-90); p<0.01).

**Table 4:** Comparison of the EQ-VAS scores between the fatigue and non-fatigue groups Missing data: EQ-VAS = 1. EQ-VAS: EuroQol 5 Dimensions 5-Level Visual Analog Scale.

	Overall (n = 72)	Fatigue (n = 46)	Non-fatigue (n = 26)	p-value
EQ-VAS (IQR)	80 (70-90)	72.5 (60-83.8)	90 (80-90)	＜0.01

**Figure 2:**
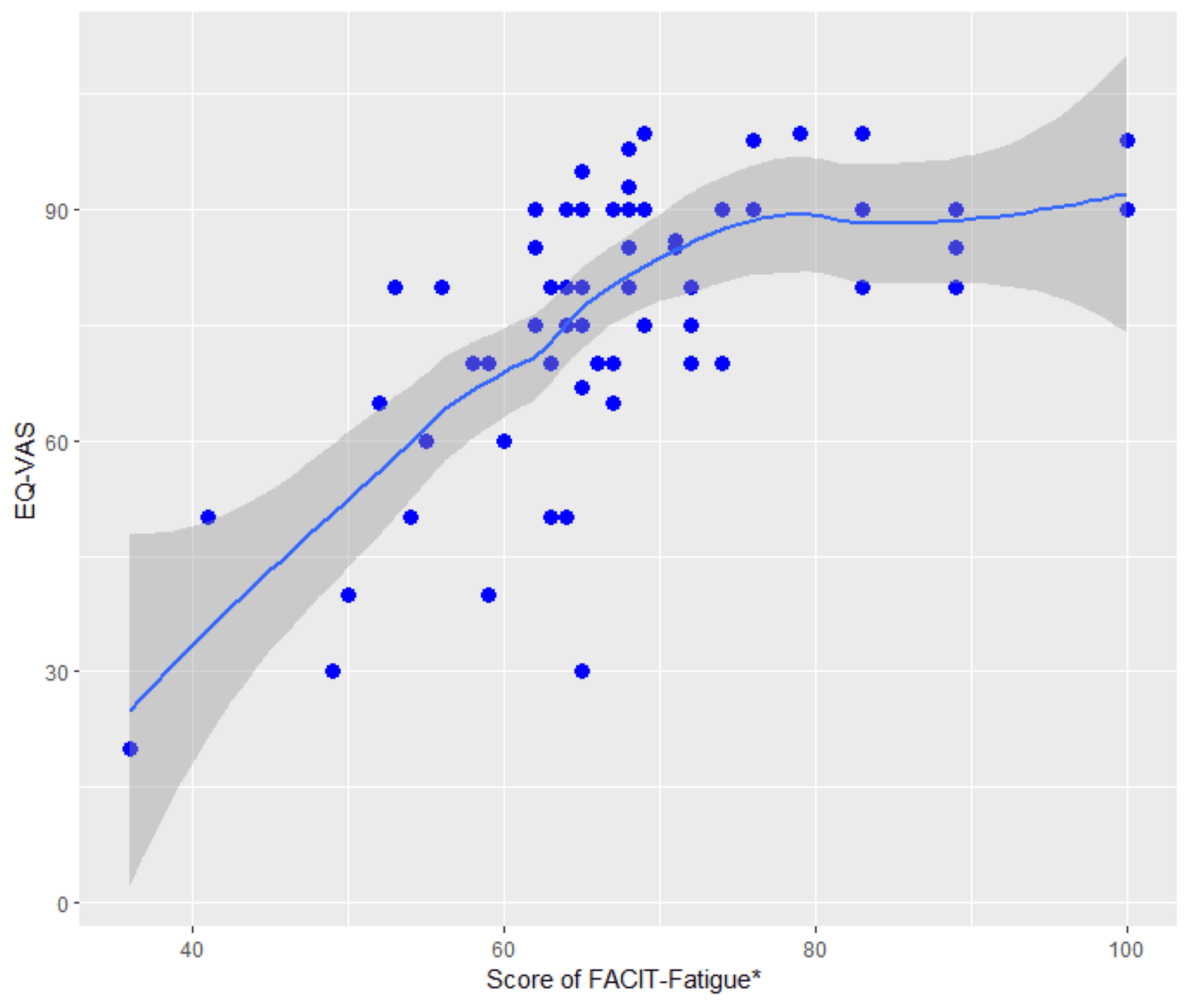
Locally weighted scatterplot smoothing (LOWESS) analysis between EQ-VAS and fatigue *Lower scores indicate a higher degree of fatigue. EQ-VAS: EuroQol 5-Dimensions 5-Level Visual Analog Scale, FACIT-F: Functional Assessment Chronic Illness Therapy-Fatigue Scale.

Figure 2 shows the association between the FACIT-F and EQ-VAS scores, indicating that lower FACIT-F scores correlated with reduced EQ-VAS scores.

## Discussion

This study investigated the prevalence of fatigue in patients six months after ICU discharge. The findings revealed that 63% of patients experienced fatigue, highlighting a significant challenge faced by ICU survivors. Additionally, BMI was identified as an independent risk factor for fatigue. Furthermore, a higher degree of fatigue was correlated with lower self-rated health among patients. These findings contribute to a better understanding of fatigue in ICU survivors and provide a foundation for future research focused on developing effective prevention and management strategies, as well as exploring the long-term impacts of fatigue on these patients.

Comparing the prevalence of fatigue in this study with those in previous studies is challenging due to differences in patient populations, follow-up periods, and assessment scales. First, many previous studies on post-ICU fatigue have focused on patients with ARDS. For example, an analysis of data from the ALTOS study [6] reported a 70% fatigue prevalence at six months and one year after ICU discharge in ARDS patients. Second, inconsistencies in the follow-up periods further complicate the comparisons. The time from ICU discharge to follow-up varies widely among studies, ranging from two weeks [18] to one year [6]. Previous studies have reported fatigue prevalence ranging from 13.8% to 80.8% [2]. For instance, a study of 2,345 patients who spent more than 12 hours in a mixed ICU found that 48% of 1,412 surgical patients reported fatigue 12 months after ICU discharge [19]. In contrast, a study examining medical-surgical ICU patients three months after discharge reported a fatigue prevalence of 15.3% [20]. Lastly, the fatigue assessment scales used varied across the studies. For example, a study assessing fatigue six months post-discharge in medical-surgical ICU patients [21] employed the Revised Piper Fatigue Scale [22] and found that 15.5% of patients experienced mild, moderate, or severe fatigue symptoms. Even within similar ICU populations, variations in assessment tools and follow-up periods have led to different prevalence rates.

While previous studies on patients with ARDS have reported high prevalence rates of fatigue, our findings indicate a similar pattern among patients in medical-surgical ICUs. Although our cohort had a shorter ICU stay and lower severity of illness than those in previous studies [6], more than half of the patients experienced fatigue. This finding may be attributable to the high proportion of older patients and patients with cardiac conditions in our study population. Previous studies have indicated that fatigue is more common among the community-dwelling older population [23]. The high prevalence of older age in the present study may have contributed to the observed fatigue rate. Additionally, patients with cardiac conditions are more likely to experience fatigue owing to factors such as heart failure pathophysiology, depression related to heart failure, and reduced social engagement [24]. Therefore, the relatively high prevalence of patients with cardiac disease in our cohort may have been associated with an increased rate of fatigue.

Higher BMI at admission may be associated with fatigue after discharge. Consistent with previous findings, a higher BMI was associated with fatigue; however, the underlying mechanism remains unclear. Studies on populations excluding ICU patients have reported that a higher BMI is a risk factor for fatigue in individuals with inflammatory bowel disease [25]. Furthermore, in patients positive for coronavirus disease 2019, elevated BMI was associated with chronic fatigue lasting more than three months [26]. Additionally, a previous study investigating the association between body fat percentage, habitual physical activity, and systemic inflammation and fatigue in healthy older adults [27] found that a higher fat percentage was associated with several aspects of fatigue, including general and physical fatigue, and reduced activity levels in older adults. Elevated BMI may contribute to decreased physical activity, increased joint stress, and reduced efficiency in daily functioning [28]. Obesity is also associated with chronic inflammatory responses that may contribute to fatigue [29]. Future investigations on factors such as inflammatory responses, physical activity, and nutritional status in relation to BMI may provide valuable insights into the underlying mechanisms of fatigue and contribute to the identification of susceptible patients.

A negative association was observed between post-ICU admission fatigue and self-rated health. The results of this study suggest that fatigue may be a contributing factor to the decline in subjective health perception. In qualitative studies investigating post-ICU fatigue, participants described the significant impact of fatigue on various aspects of life, including physical, social, cognitive, and emotional domains, highlighting the complex interactions among these factors [30]. For instance, some participants reported that fatigue affected their ability to be socially active or work as they had before their critical illness. These findings indicate that post-ICU fatigue is associated with multiple aspects of daily life. Fatigue involves a decrease in physical and mental health, which may lead to difficulties in daily activities and reduced activity levels, ultimately contributing to a more negative perception of overall health. Given the multifactorial nature of fatigue, our findings underscore the need for comprehensive support, including psychological care, social support, and health education, for patients following ICU discharge.

Strength and limitations 

To the best of our knowledge, this is the first study in Japan to investigate the prevalence of fatigue after ICU discharge using a scale with confirmed validity and reliability. Previous studies have explored fatigue following ICU discharge using various scales, and this study used the FACIT-F, the reliability and validity of which have been validated, specifically in post-ICU patient populations. From this perspective, we consider our study results valid. However, this study had several limitations. First, we did not assess fatigue before ICU admission. Therefore, the extent of fatigue experienced by patients before ICU admission remains unclear. Second, as most of our participant admissions were scheduled and this was a single-center study, external validity may be limited. However, data from the Japanese Intensive Care Patient Database [4] indicate that 63% of ICU admissions in Japan are scheduled, with cardiovascular disease accounting for 26.2% of these cases. This finding was similar to that observed in our study population. Therefore, our results may reflect the ICU admission population admitted to the ICUs in Japan. Third, the association between unmeasured variables and post-ICU admission fatigue remains unclear. Fourth, the primary aim of this study was to estimate the overall prevalence of post-ICU fatigue, and the sample size calculation was tailored to this purpose, which limited the sample size. In future studies, a larger sample size would enable us to examine potential subgroup differences that may not have been detected in this study. Fifth, since the risk factors were studied in an exploratory manner, further investigation is needed to identify those associated with fatigue. Sixth, the excluded patient population may include individuals experiencing fatigue, potentially leading to an underestimation of the results. Seventh, our study was a single-center investigation conducted over six months. Further multicenter studies with larger samples and patients from diverse backgrounds are warranted. This will enhance the generalizability of the findings and enable the derivation of conclusions applicable to a broader population. Eighth, it is also possible that fatigue itself is inherently considered a component of subjective health perception, which could influence the observed association. In the future, it is crucial to conduct comprehensive studies that evaluate the physiological and psychological aspects of fatigue, its impact on QOL, and its interactions with other health-related factors.

Clinical implications

Few studies have examined the effectiveness of treatments or care for patients with fatigue following ICU discharge [3]. In Japanese ICUs, a significant proportion of patients are older and admitted due to cardiac conditions, which may predispose them to experiencing fatigue. This highlights the growing need for a deeper understanding of patient distress and the importance of follow-up care. Routine evaluations of fatigue during ICU stays and follow-up visits could help clinicians identify patients at risk of prolonged recovery. These assessments could then inform personalized interventions, such as tailored physical rehabilitation programs, psychological support, or adjustments in medication regimens. As part of PICS prevention and care, a multifaceted approach tailored to each patient's symptoms, combined with ongoing follow-up, is essential for effectively managing fatigue.

Research implications

Assessing fatigue before ICU admission and tracking long-term changes after ICU discharge are important for understanding fatigue among ICU survivors. Additionally, it would be beneficial to use standardized scales across studies. Future research should aim to assess the risk factors for post-ICU fatigue in greater detail using larger sample sizes.

## Conclusions

This study demonstrated that a significant number of patients in Japanese ICUs experience fatigue, even when their disease severity is low. Additionally, BMI at ICU admission was identified as an independent risk factor for fatigue six months after ICU discharge. These findings provide a foundation for future research focused on developing effective strategies for preventing and managing fatigue, as well as understanding its long-term impact on ICU survivors. Regular follow-up on fatigue after ICU discharge is essential for improving long-term outcomes.
